# The Relationship between Cognitive Dysfunction and Postural Stability in Multiple Sclerosis

**DOI:** 10.3390/medicina58010006

**Published:** 2021-12-21

**Authors:** Justyna Redlicka, Ewa Zielińska-Nowak, Anna Lipert, Elżbieta Miller

**Affiliations:** 1 Department of Neurological Rehabilitation, Medical University of Lodz, Milionowa 14, 93-113 Lodz, Poland; justyna.redlicka@umed.lodz.pl (J.R.); ewa.zielinska@umed.lodz.pl (E.Z.-N.); 2 Department of Sports Medicine, Medical University of Lodz, Pomorska 251, 92-213 Lodz, Poland; anna.lipert@umed.lodz.pl

**Keywords:** multiple sclerosis, fatigue, postural stability, balance, disability, cognitive function

## Abstract

*Background and Objectives*: Multiple Sclerosis (MS) is a demyelinating disease of the central nervous system (CNS), most commonly characterized by balance dysfunction, fatigue syndrome and cognitive impairment. The goal of our study was to determine the association between cognitive functions and static posture control. *Materials and Methods*: The research group consisted of 76 randomized MS patients (ICDG 35.0) hospitalized at the Neurological Rehabilitation Clinic of the Medical University of Lodz. This group was divided into three subgroups according to the cognitive assessment based on the Mini Mental State Examination (MMSE) for patients over 65 years of age and the Montreal Cognitive Assessment (MoCA) under the age of 65. Fatigue syndrome was assessed using the Fatigue Severity Scale (FSS), and postural stability using a stabilometric platform. *Results:* The men demonstrated poorer stabilometric platform measurements than the women. Statistically significant differences were observed between patients without dysfunction and severe cognitive impairment. The results of the stabilometric platform were found to correlate with body mass index in all three groups of patients (Spearman’s test). *Conclusions*: Body mass index and cognition have impact on postural stability in MS patients with moderate disability and fatigue syndrome.

## 1. Introduction

Multiple sclerosis (MS) is a chronic disease of the central nervous system (CNS), with more than half of patients demonstrating cognitive dysfunction, while only 2% presented experience-blunted intellectual functioning. Cognitive decline was also indicated as one of the main symptoms of MS by Jean Martin Chacort in his comprehensive description of MS in 1868 [1]. Currently, it is well known that cognitive dysfunction reduces the everyday activity of MS patients and can decrease quality of life [2], and is a major reason for early retirement. It may also have an impact on fatigue syndrome, which is reported in 70–80% of MS patients. Generally, fatigue can be influenced in the motoric, psychosocial, and cognitive dimension [3]. Cognitive fatigue has also been found to significantly impair daily life and accelerate motoric fatigue [4].

Data indicate that MS patients most commonly demonstrate problems in cognitive processing speed and episodic memory. The most frequently observed cognitive problems include deficits in information processing speed, episodic memory, complex attention, and executive function [5,6,7]. Cognitive impairment can be observed even in the early stages of the disease [8,9]. Brain imaging clinical studies indicate a link between the severity of cognitive impairment and the loss of brain volume or brain atrophy (neurodegeneration feature). Such neurodegeneration is more characteristic of the chronic stage of disease and gradually leads to a progressive disability; it has a profound impact on the quality of life of people with MS [10].

Among the symptoms influencing functional state, balance, and core stability disturbances [11], paresis (mainly in the lower limbs) [12] and fatigue [13] are believed to play prominent roles [14,15,16]. However, it is still not clear which of these symptoms should be prioritized to optimize rehabilitation. Postural stability is believed to be influenced by a range of factors including cognitive processing, emotional status, visual feedback, and cerebellar activity. Problems in any of the mechanisms involved in postural control can have an impact on balance and postural sway and can interfere with the frequency of accidental falls and injuries. Moreover, postural control in MS can be disrupted not only by impairments in the integration and function of neural pathways in CNS, but also by damage in the peripheral organs which also act as sensory receptors. Clinical studies suggest that severe disability, a chronic–progressive form of disease, worse postural control and cognitive dysfunction are particularly associated with a higher level of accidental falls.

Objective estimations of balance based on automatic and standardized assessments can be performed using static posturography [17,18]. Such analyses based on laboratory-graded force platforms can yield a number of parameters, the most important and useful of which is Center of Pressure (COP). The results of posturographic clinical studies have been found to correlate with disability status assessed in the Expanded Disability Status Scale (EDSS) [19] and severity of cognitive impairment. However, the clinical estimation of MS patients is difficult due to the complex course of disease, with many additional medical problems influencing the final patient review. Currently, the introduction of novel therapies in MS should be much more individually tailored.

Therefore, the aim of this study is to make a complex estimation of static postural control parameters in a very specific group of MS patients (moderately disabled; with fatigue syndrome), labeled MS-MD-F. This group was subdivided into three groups to compare participants without any cognitive dysfunction, those with mild cognitive impairment and those with severe cognitive problems. The study also identifies which postural control parameters are the highest out of proper rate in the MS-MD-F group and estimates their impact on cognitive impairment level. It also presents a correlation between postural stability parameters and age, body mass index (BMI), and fatigue syndrome.

## 2. Materials and Methods

### 2.1. Participants

The study group consists of 76 randomized MS patients (ICD G35.0) hospitalized at the Neurological Rehabilitation Department of the Medical University of Lodz, Poland. The patient was followed for 4 weeks. The inclusion criteria for this study were the following: diagnosis of MS according to the McDonald criteria; age 18 years or older; EDSS score between 3,5–6; diagnosis of fatigue syndrome based on FSS score over 38; lack of relapses during the previous three months. Based on previous studies [20,21], the MS-MD-F study group was stratified into three subgroups according to cognitive assessment on the Montreal Cognitive Assessment (MoCA) and Mini-Mental State Examination (MMSE) scales. MS-MD-F0 (MMSE ≥ 27/MoCA ≥ 26) without cognitive impairment; MS-MD-F1 (18 ≤ MMSE ≤ 26/25 ≤ MoCA ≤ 10) mild cognitive impairment; MS-MD-F2 (MMSE ≥ 17/MoCA ≥ 10) severe cognitive impairment. All MMSE/MoCA assessments performed by the patient’s psychologist.

Demographic and clinical descriptive data were obtained from medical records (Table 1). The experimental procedure was approved by the Local Research Ethics Committee according to the Helsinki Declaration and were approved by the Ethics Committee of the Medical University of Lodz, Poland, RNN/168/21/KE. This study was conducted by an experienced rehabilitation team: medical doctor—enrollment, supervisor; physiotherapist—stabilometric assessment, estimation of EDSS scale; psychologist—estimation in psychological scales).

### 2.2. Clinical Scales

Baseline data included age, sex, MS diagnosis ICD (G 35.0), body mass index (BMI), body mass, and height (Table 1).

Regarding education, no participants reported only primary or vocational education (7–11 years of education); all had completed intermediate (secondary education: 12–13 years) or higher education (university education: 15–17 years). Fatigue syndrome was assessed using the Fatigue Severity Scale (FSS), which is a validated tool (nine fatigue-related questions rated on a seven-point scale). To include only fatigued patients, a cut-off value 38 was used, as suggested by Flachenecker et al. [22]. The EDSS is the most commonly used scale for the assessment of functional status impairment and disability [23]. The range of the EDSS scale was selected due to the requirements of the study. We also used this range in our earlier study [24]. The patient had to stand up, either alone or with orthopedic assistance, for 2 × 30 s and could move even in a small area. Among researchers, the use of scales to assess cognition by age can be observed [24]. Cognitive functioning was assessed with the MMSE in MS patients over 65 years old and with the MoCA below the age of 65. MMSE is often used to assess dementia or Alzheimer’s disease [25,26]. Both scales are the most widely used screening tests in people with CNS diseases, including MS, and provide a brief and objective measure of cognitive functioning.

### 2.3. Stabilometric Assessment

Postural control was assessed using the CQStab2P stabilometric platform in a two-plate version (CQ Elektronik System). Before the measurement, the platforms were balanced and leveled. The study participant stood on a platform with strain gauges placed in the corners, recording the central pressure of the feet on the ground, as well as its movements in the sagittal X (left–right) and frontal (anterior–posterior) axes. The projection of the COP of the feet on the ground was recorded as a point and as a dynamic parameter that changes its position per unit of time. This test was carried out with eyes opened and closed after 30 s. The results of the study are presented in the form of graphs called a statokinesiogram and stabilogram.

To ensure safety during the study, the patients were protected by a physiotherapist, without disturbing the course of the study.

Selected parameters of the COP of the feet were analyzed:

The mean deflection of the COP of the feet from point 0 in the direction of the Y axis (eyes opened/eyes closed) MAAP-EO/EC [mm], the mean deflection of the COP of the feet from point 0 in the direction of the X axis (eyes opened/eyes closed)—MAML-EO/EC [mm], median difference balance (eyes opened/eyes closed; right and left leg) MDDB-EO/EC R L, total path length measured in both axes (2D) (eyes opened/eyes closed) SP-EO/EC [mm], the length of the statokinesiogram path in the direction of the Y axis (eyes opened/eyes closed) SPAP-EO/EC [mm], the length of the statokinesiogram path in the direction of the X axis (eyes opened/eyes closed) SPML-EO/EC [mm], the number of COP deflections in the Y axis (eyes opened/eyes closed) LWAP EO/EC, the number of COP deflections in the X axis (eyes opened/eyes closed) LWML EO/EC.

### 2.4. Statistical Analysis

Statistical analyses were performed using Statistica version 13.1 software (StatSoft). The data were not normally distributed, so non-parametric tests were used. The statistical analysis was performed by Mann–Whitney U-test t for two independent variables or ANOVA for more than two independent variables. The effect size, measuring the differences between the results inside the groups and between groups, was determined by Cohen’s d; the value is defined as the difference between two means divided by a standard deviation for the data. Effect sizes were recorded as small (d = 0.2), medium (d = 0.5), and large (d ≥ 0.8). Spearman’s correlation coefficient was used to assess the relationship between the results of stabilometric platform/static postural control and age and body mass. For all analyses, significant differences were accepted at the level of *p* < 0.05.

## 3. Results

### 3.1. Stabilometric Platform Results/Static Postural Control

MS-MD-F1 and MS-MD-F2 patients sometimes achieved poorer stabilometric platform measurement results than the MS-MD-F0 patients. Statistically significant differences were observed between MS-MD-F0 and MS-MD-F2 patients with regard to MAML-EO1, MDDB-EC1 L and SP-EC5, with moderate effect size, and in SPML-EO5, with small effect size. Statistically significant differences were also observed between MS-MD-F0 and MS-MD-F1 patients with regard to MDDB-EO1 R, SP-EC1, SP-EC3, and SP-EC5, with moderate effect size, and in SP-EO1 and SPML-EO6, with small effect size (Table 2).

Generally, the male participants obtained poorer stabilometric scores than the female participants, with statistically significant differences observed in SPAP-EC between the male and female MS-MD-F0 members, and in LWML-EC between the male and female MS-MD-F1 members (Table 3). However, significantly poorer LWML-EC scores were noted in both men and women from MS-MD-F2 than those from MS-MD-F0 and MS-MD-F1 (Table 3).

### 3.2. Spearman Correlation

Spearman’s correlation test identified a relationship between the results of stabilometric platform and age, but the results are not statistically significant. A positive relationship was observed between static postural control and age in MS-MD-F2 patients in LWML-EO10 and MDDB-EO1 R. In MS-MD-F2 patients, a negative relationship was observed between age and MAAP-EO1 and MDDB-EO1 L.

Spearman’s test showed a relationship between stabilometric platform assessment and body mass in all the three groups of patients. In the MS-MD-F0 group, the strongest positive and statistically significant relationship was observed with SP-EC3, SP-EC5, and SP-EC9. In MS-MD-F1 patients, the strongest positive relationship was observed with SP-EC9. In MS-MD-F2 patients, the strongest positive and statistically significant relationships were observed with MDDB-EO1 R and MDDB-EC1 R. In MS-MD-F2 patients, the strongest negative and statistically significant relationship was observed with MDDB-EO1 L and MDDB-EC1 L (Table 4).

Spearman’s test also showed some relationship between the results of stabilometric platform evaluation and body mass according to sex. In general, among men, a positive and statistically significant relationship was observed with LWML-EC, while those in the MS-MD-F0 demonstrated a significant correlation with SPAP-EC. In female MS-MD-F0 members, a positive relationship was observed for MDDB-EO R and a negative relationship was observed for SPML-EO and MDDB-EO L (Table 5).

## 4. Discussion

Posturographic testing is the basic objective method of postural stability assessment. The last standardization protocol for posturography by the International Society of Posturography was published in 1983. Therefore, there is a need to update this information to account for novel therapies in MS rehabilitation [27,28]. Analysis of COP movements provides a wealth of information about postural stability. Based on the recorded data, it is possible to assess the variability of body deflection when maintaining balance in a standing position and this assessment is particularly important in conditions of disturbed postural stability [29].

Symptomatic fatigue is significantly related to balance and in persons with MS, it can act as a significant predictor of balance as a function of central sensory integration. Fatigue and balance are associated with cerebellar and brainstem involvement. This study provides information about the frequency of cognitive deficits in specific group of MS patients with fatigue syndrome and a moderate/severe level of disability.

Firstly, from the studied group of 76 participants, over 60% presented mild cognitive impairment, 27% did not demonstrate any cognitive dysfunction, and 11% presented severe cognitive problems. It has already been stated that about 40 to as many as 70% of patients with MS suffer from cognitive impairment [30].

Problems with postural control and cognitive dysfunctions are common symptoms in MS patients. Accumulating data suggest that postural and cognitive tasks may interfere with each other when performed concomitantly [31].

Therefore, it has been hypothesized that a link exists between the postural control and cognitive domains [32,33], defined as cognitive–motor interference/cognitive–posture interference.

Currently, the key therapy for improving postural control and balance in MS is rehabilitation [34].

No statistically significant differences in stabilometric parameters were found between the MS-MD-F0 group and the entire population of 76 studied patients (MS-MD-F). However, patients with mild cognitive dysfunction demonstrated changes in mainly MDDB EO, SP-EO1, and SP-EC1,3,5 in comparison to those without any cognitive problems. Mild cognitive impairment (MCI) has been found to affect specific gait parameters and static balance, suggesting that both parameters may be useful for diagnosis and outcome analysis [35]. This may be a valuable finding as up to 50–80% of patients experience balance and gait disturbances, of whom 50% experience a fall at least once a year [36]

The BMI estimations in our study indicate 35% of MS participants to be overweight (score rate: 25–29.9 kg/m^2^) and 18% to demonstrate first-degree obesity (score rate: 30–34.9 kg/m^2^). Comparable scores were found in a study of a typical Polish population, comprising 14 537 persons (aged 20–74 years); 18.9% of men, and 18.0% of women demonstrated class I obesity while 43.2% of men and 30.5% of women were overweight [37]. However, a relationship was noted between the stabilometric platform data and BMI in all three groups of MS patients. Therefore, BMI is a factor that might have an influence on postural stability in MS patients together with the level of disability and additional medical problems including fatigue syndrome and cognitive impairment [38]. Clinical studies reported that obese people presented greater COP displacement at higher AP velocities compared to non-obese people, which suggests that obese subjects are predisposed to worse balance control [39].

Generally, many stabilometric studies have presented that MS patients have significantly poorer postural sway control than healthy subjects, demonstrating larger oscillations in both the frontal and sagittal planes [40,41]. Posturographic research by Morrison et al. found that persons with MS presented greater overall COP motion in both the medio-lateral (ML) and anterior–posterior (AP) directions compared to older adults. Moreover, during more difficult balance workouts, people with MS presented more significant ML than AP motion [42].

However, this statement is not in line with our study findings. The present participants formed a very specific group of MS patients with fatigue syndrome and moderate level of disability. Our present analysis identified the presence of statistically significant differences in four parameters of postural stability between groups with mild and severe cognitive impairment. Differences in SPML-EO6 were found to be specific for cognitive disfunction for both mild and severe cognitive dysfunction.

These findings offer support for the existence of a strong relationship between age and postural control. The degree of medio-lateral (ML) sway appeared to play an important role in predicting balance ability. It would also seem that women have better postural control than men. A similar impact of sex on postural stability, i.e., a slight tendency toward better balance in women, has been noted in previous studies [43,44,45]. This improved postural control may be achieved by the use of the ankle strategy, or in the case of severe body balance problems, by the hip strategy [27]. Clearly there is a need to update the current standardization protocol for posturography, published by the International Society of Posturography in 1983 [28].

The strength of the study is that it uses a study group including patients with different cognitive impairment degree; this made it possible not only to characterize static postural control in general, but also to present the main differences between the groups more clearly. The results have clinical relevance in the care of MS patients because they can help direct further interventions to improve postural control and target approaches for gait interventions.

However, some limitations have to be mentioned. Firstly, only a few people with MS could be included in the study, especially those with severe cognitive impairment. Secondly, the study participants were recruited from only one Division at the University. Different additional characteristics, such as MS lesion locations or medication in use, could affect the results. However, there is no sufficient evidence confirming the relationship between drugs or MS lesion location on cognitive function in MS. Additionally, it would be worth conducting future research analyzing the impact of physical training not only on cognition as a whole, but in separation in the spheres which constitute the cognitive function. Further studies, including more medical centers, are needed to explore the generalizability of our findings.

## 5. Conclusions

Clinical research with MS patients is complicated by the variety of clinical types, dynamic changes in severity of patients’ symptoms, and the unpredictable course of the disease. As such, treatment and rehabilitation in everyday medical practice is beset with many unsolved problems. The clinical picture of a patient with MS is also affected by additional impairments including cognitive dysfunction, fatigue syndrome, and degree of disability. Therefore, a new broader and more individually tailored therapeutic approach focusing on specific groups of patients seems to be beneficial.

## Figures and Tables

**Table 1:** Baseline data.

	MS-MD-F	MS-MD-F0	MS-MD-F1	MS-MD-F2
AGE (YEARS)	55.54 ± 13.24	50.05 ± 17.62	57.04 ± 10.95	59.50 ± 8.33
BODY MASS (KG)	76.59 ± 14.45	75.38 ± 15.63	77.03 ± 14.88	76.71 ± 10.32
HEIGHT (CM)	169.84 ± 9.20	168.76 ± 8.51	170.35 ± 8.87	171.75 ± 12.16
BMI (KG/CM^2^)	25.91 ± 4.71	26.38 ± 3.81	25.44 ± 5.06	26.37 ± 4.47
FSS	41.26 ± 7.38	41.33 ± 5.77	40.53 ± 7.90	43.87 ± 7.70
MALE/FEMALE (N)	36/40	9/13	24/22	4/4

FSS: Fatigue Severity Scale; MS-MD-F—multiple sclerosis with moderate disability and fatigue (0—without cognitive impairment; 1—mild cognitive impairment; 2—severe cognitive impairment).

**Table 2:** The differences in stabilometric platform results between different levels of cognitive impairment in multiple sclerosis patients with moderate disability and fatigue syndrome.

	MS-MD-F (n = 76)	MS-MD-F0 (n = 22)	MS-MD-F1 (n = 46)	MS-MD-F2 (n = 8)
MAAP-EO	5.28 ± 3.79	5.38 ± 3.76	5.30 ± 3.85	5.29 ± 4.05
MAML-EO	4.29 ± 4.70	5.30 ± 6.05	4.13 ± 4.27	2.90 ± 2.94 *^,M^
MDDB-EO L	48.32 ± 9.22	45.76 ± 8.50	49.93 ± 10.02	46.50 ± 3.62
MDDB-EO R	51.68 ± 9.22	54.24 ± 8.50	50.06 ± 10.02 ^#,M^	53.50 ± 3.62
MAAP-EC	5.21 ± 4.09	5.39 ± 5.58	5.21 ± 3.27	5.09 ± 4.48
MAML-EC	3.52 ± 4.31	3.79 ± 5.59	3.53 ± 3.95	3.06 ± 2.74
MDDB-EC L	48.86 ± 9.23	48.10 ± 8.36	49.76 ± 10.19	46.75 ± 4.65 *^,M^
MDDB-EC R	51.14 ± 9.23	51.90 ± 8.36	50.24 ± 10.19	53.25 ± 4.65
SP-EO	401.59 ± 271.08	430.14 ± 323.75	381.37 ± 244.89 ^#,S^	430.37 ± 307.93
SPAP-EO	273.85 ± 177.50	289.33 ± 210.69	257.50 ± 153.36	309.25 ± 229.48
SPML-EO	222.18 ± 194.76	245.14 ± 233.69	213.19 ± 183.85	216.75 ± 175.59 *^,S^
LWAP-EO	15.97 ± 8.68	16.24 ± 9.78	15.43 ± 8.34	18.12 ± 8.85
LWML-EO	14.13 ± 8.15	13.57 ± 8.74	13.94 ± 6.94	16.62 ± 13.24
SP-EC	413.57 ± 420.95	453.28 ± 529.72	374.92 ± 368.26 ^#,M^	392.25 ± 180.32 *^,S^
SPAP-EC	321.97 ± 319.77	329.24 ± 378.63	291.91 ± 281.91 ^#,M^	352.12 ± 168.03 *^,S^
SPML-EC	180.50 ± 221.53	225.81 ± 320.46	159.35 ± 175.86 ^#,M^	139.25 ± 69.72 *^,M^
LWAP-EC	19.45 ± 13.89	16.67 ± 12.12	20.09 ± 15.21	20.87 ± 9.39
LWML-EC	17.64 ± 12.20	15.71 ± 13.14	17.28 ± 11.72	23.87 ± 12.43

MS-MD-F—multiple sclerosis with moderate disability and fatigue (0—without cognitive impairment; 1—mild cognitive impairment; 2—severe cognitive impairment); MAAP: mean COP displacement from point 0 in the Y axis direction; EO: eyes open; MAML: mean COP displacement from point 0 in the X axis direction; MDDB: EC: eyes closed; SP: total path length on the XY axes; SPAP: path length measured on the Y axis direction; SPML: path length measured on the X axis direction; LWAP: number of COP displacements along the Y axis; LWML: number of COP displacements along the X axis; significant differences between MS-MD-F2 and MS-MD-F0 (* *p* < 0.05), and between MS-MD-F1 and MS-MD-F0 (^#^ *p* < 0.05). The letters ^M^ and ^S^ indicate moderate and small size effects.

**Table 3:** The differences in stabilometric platform results between different levels of cognitive impairment in multiple sclerosis males and females with moderate disability and fatigue syndrome.

	MS-MD-F		MS-MD-F0		MS-MD-F1		MS-MD-F2	
	MALES (N = 36)	FEMALES (N = 40)	MALES (N = 9)	FEMALES (N = 13)	MALES (N = 24)	FEMALES (N = 22)	MALES (N = 4)	FEMALES (N = 4)
**MAAP-EO**	5.68 ± 4.36	4.91 ± 3.21	4.79 ± 3.03	5.75 ± 4.21	5.89 ± 4.68	4.65 ± 2.64	6.22 ± 5.37	4.35 ± 2.66
**MAML-EO**	5.47 ± 6.08	3.23 ± 2.62	7.62 ± 8.86	3.87 ± 3.07	4.93 ± 5.33	3.25 ± 2.51	4.40 ± 3.68	1.40 ± 0.78
**MDDB-EO L**	49.61 ± 10.49	47.15 ± 7.87	48.87 ± 3.83	43.85 ± 10.06	50.21 ± 12.60	49.63 ± 6.43	47.50 ± 4.72	45.50 ± 2.38
**MDDB-EO R**	50.39 ± 10.49	52.85 ± 7.87	51.12 ± 3.83	56.15 ± 10.06	49.79 ± 12.60	50.36 ± 6.43	52.50 ± 4.72	54.50 ± 2.38
**MAAP-EC**	5.23 ± 3.29	5.20 ± 4.73	4.39 ± 2.59	6.01 ± 6.85	5.84 ± 3.60	4.52 ± 2.78	3.27 ± 0.84	6.90 ± 6.11
**MAML-EC**	3.73 ± 4.12	3.32 ± 4.50	3.70 ± 3.51	3.84 ± 6.70	3.94 ± 4.63	3.09 ± 3.09	2.52 ± 1.70	3.60 ± 3.72
**MDDB-EC L**	49.90 ± 10.92	47.92 ± 7.40	48.25 ± 3.73	48.04 ± 10.40	50.68 ± 13.22	48.76 ± 5.39	48.50 ± 2.08	45.00 ± 6.16
**MDDB-EC R**	50.10 ± 10.92	52.08 ± 7.40	51.75 ± 3.73	51.99 ± 10.40	49.31 ± 13.22	51.24 ± 5.39	51.50 ± 2.08	55.00 ± 6.16
**SP-EO**	440.31 ± 306.97	366.75 ± 232.63	509.62 ± 350.83	381.23 ± 309.98	409.04 ± 283.56	351.18 ± 196.49	489.25 ± 414.59	371.50 ± 200.31
**SPAP-EO**	304.91 ± 210.36	245.90 ± 138.50	389.37 ± 268.04	227.77 ± 145.91	274.58 ± 178.30	238.86 ± 121.99	318.00 ± 279.79	300.50 ± 210.69
**SPML-EO**	242.99 ± 200.21	203.45 ± 190.29	243.12 ± 202.95	246.38 ± 258.82	234.81 ± 203.03	189.59 ± 161.76	291.75 ± 227.62	141.75 ± 71.66
**LWAP-EO**	17.72 ± 9.51	14.40 ± 7.64	18.25 ± 13.96	15.00 ± 6.44	17.33 ± 8.51	13.36 ± 7.82	19.00 ± 6.38	17.25 ± 11.84
**LWML-EO**	15.56 ± 9.42	12.85 ± 6.66	15.87 ± 10.79	12.15 ± 7.32	14.51 ± 6.91	13.32 ± 7.07	21.25 ± 18.59	12.00 ± 2.58
**SP-EC**	492.10 ± 518.72	342.90 ± 297.45	783.37 ± 740.71	250.15 ± 174.03	413.69 ± 448.71	332.64 ± 257.87	380.00 ± 152.12	404.50 ± 228.75
**SPAP-EC**	386.36 ± 387.68	264.02 ± 233.44	574.62 ± 519.42	178.23 ± 129.26 ^b,M^	328.37 ± 356.07	252.14 ± 168.08	357.75 ± 171.37	346.50 ± 190.85
**SPML-EC**	229.42 ± 295.60	136.47 ± 108.44	386.62 ± 476.86	126.84 ± 103.26	193.01 ± 227.48	122.64 ± 83.01	133.50 ± 57.61	145.00 ± 89.08
**LWAP-EC**	22.48 ± 16.61	16.72 ± 10.37	21.12 ± 14.49	13.92 ± 10.04	23.55 ± 18.29	16.32 ± 10.05	18.75 ± 11.47	23.00 ± 7.87
**LWML-EC**	20.61 ± 13.76	14.97 ± 10.05	21.25 ± 17.53	12.31 ± 8.70	20.83 ± 13.38	13.41 ± 8.26 ^b,S^	18.00 ± 10.49*^,a,M^	29.75 ± 12.58 *^,a,M^

The significant differences between the results: SEVERE vs. HEALTHY. * *p* < 0.05. MILD vs. HEALTHY *p* < 0.05 SEVERE vs. MILD ^a^ *p* < 0.05 MALES VS. FEMALES ^b^ *p* < 0.05. The letters ^M^ and ^S^ indicate the size effect of moderate and small, respectively.

**Table 4:** Correlations between the results of stabilometric platform/static postural control and body mass among the participants with different cognitive disfunctions.

Variables	Spearman’s Correlation			
	MS-MD-F	MS-MD-F0	MS-MD-F1	MS-MD-F2
**SP-EO**	0.050	0.106	−0.035	0.476
**SPAP-EO**	0.118	0.327	0.016	0.333
**SPML-EO**	0.019	−0.067	−0.003	0.452
**LWAP-EO**	0.065	0.041	0.059	0.275
**LWML-EO**	0.073	0.330	−0.067	0.108
**SP-EC**	0.108	0.370	−0.070	0.167
**SPAP-EC**	0.125	0.457	−0.083	−0.095
**SPML-EC**	0.172	0.413	−0.014	0.619
**LWAP-EC**	0.251	0.472	0.070	0.667
**LWML-EC**	0.347	0.436	0.302	0.667
**MAAP-EO**	0.040	−0.234	0.119	0.405
**MAML-EO**	0.167	0.150	0.212	−0.191
**MDDB-EO L**	−0.033	−0.081	0.013	−0.786
**MDDB-EO R**	0.034	0.081	−0.013	0.786
**MAAP-EC**	0.002	−0.181	0.037	0.371
**MAML-EC**	0.074	0.288	−0.081	0.467
**MDDB-EC L**	−0.017	−0.042	0.072	−0.754
**MDDB-EC R**	0.017	0.042	−0.073	0.754

MS-MD-F—multiple sclerosis with moderate disability and fatigue (0—without cognitive impairment; 1—mild cognitive impairment; 2—severe cognitive impairment).

**Table 5:** Sex differences in the correlations between the results of stabilometric platform/static postural control and body mass in MS patients.

Variables	Spearman’s Correlation							
	MS-MD-F		MS-MD-F0		MS-MD-F1		MS-MD-F2	
	MALES (N = 36)	FEMALES (N = 40)	MALES (N = 9)	FEMALES (N = 13)	MALES (N = 24)	FEMALES (N = 22)	MALES (N = 4)	FEMALES (N = 4)
**SP-EO**	−0.037	0.001	0.548	−0.390	−0.199	0.023	0.001	0.800
**SPAP-EO**	−0.018	0.175	0.595	−0.016	−0.183	0.151	0.600	0.400
**SPML-EO**	−0.040	−0.195	0.619	−0.582	−0.145	−0.151	0.001	1.000
**LWAP-EO**	0.075	−0.054	0.587	−0.305	0.030	−0.133	0.800	0.105
**LWML-EO**	−0.057	0.090	0.503	0.208	−0.193	−0.025	0.800	−0.200
**SP-EC**	0.049	0.021	0.667	−0.165	−0.109	−0.121	0.600	0.001
**SPAP-EC**	0.035	0.068	0.738	−0.049	−0.115	−0.150	0.001	0.001
**SPML-EC**	0.122	0.048	0.667	−0.038	−0.121	−0.091	1.000	0.600
**LWAP-EC**	0.216	0.206	0.671	0.328	0.077	−0.138	0.600	0.800
**LWML-EC**	0.369	0.190	0.595	0.377	0.292	−0.072	0.800	0.800
**MAAP-EO**	−0.187	0.106	−0.120	−0.281	−0.295	0.368	0.200	0.200
**MAML-EO**	0.226	−0.041	0.238	0.148	0.197	0.010	0.400	−0.600
**MDDB-EO L**	−0.104	−0.128	0.182	−0.689	−0.194	0.285	−0.949	−0.632
**MDDB-EO R**	0.104	0.128	−0.182	0.689	0.194	−0.285	0.949	0.632
**MAAP-EC**	−0.160	0.067	−0.190	−0.060	−0.211	0.067	0.949	0.200
**MAML-EC**	−0.115	0.204	0.287	0.355	−0.299	0.051	0.800	0.001
**MDDB-EC L**	−0.092	−0.056	0.144	−0.135	−0.171	0.345	−0.400	−0.632
**MDDB-EC R**	0.092	0.056	−0.144	0.135	0.171	−0.345	0.400	0.632

>MS-MD-F—multiple sclerosis with moderate disability and fatigue (0—without cognitive impairment; 1—mild cognitive impairment; 2—severe cognitive impairment).
